# InTiCAR: Network-based identification of significant inter-tissue communicators for autoimmune diseases

**DOI:** 10.1016/j.csbj.2025.01.003

**Published:** 2025-01-10

**Authors:** Kwansoo Kim, Manyoung Han, Doheon Lee

**Affiliations:** Department of Bio and Brain Engineering, KAIST, Daejeon 34141, Republic of Korea

**Keywords:** Inter-tissue communicators, Network analysis, Random walk with restart, Autoimmune diseases

## Abstract

Inter-tissue communicators (ITCs) are intricate and essential aspects of our body, as they are the keepers of homeostatic equilibrium. It is no surprise that the dysregulation of the exchange between tissues are at the core of various disorders. Among such conditions, autoimmune diseases (AIDs) refer to a collection of pathological conditions where the miscommunication drives the immune system to mistakenly attack one's own body. Due to their myriad and diverse pathophysiologies, AIDs cannot be easily diagnosed or treated, and continuous efforts are required to seek for potential diagnostic markers or therapeutic targets. The identification of ITCs with significant involvement in the disease states is therefore crucial. Here, we present InTiCAR, Inter-Tissue Communicators for Autoimmune diseases by Random walk with restart, which is a network exploration-based analysis method that suggests disease-specific ITCs based on prior knowledge of disease genes, without the need for the external expression data. We first show that distinct ITC profile s can be acquired for various diseases by InTiCAR. We further illustrate that, for autoimmune diseases (AIDs) specifically, the disease-specific ITCs outperform disease genes in diagnosing patients using the UK Biobank plasma proteome dataset. Also, through CMap LINCS dataset, we find that high perturbation on the AIDs genes can be observed by the disease-specific ITCs. Our results provide and highlight unique perspectives on biological network analysis by focusing on the entities of extracellular communications.

## Highlights

• InTiCAR is a prior knowledge-based *in silico* network analysis to find disease-specific inter-tissue communicators (ITCs).
• The ITCs for autoimmune diseases were useful in disease diagnoses.
• The ITCs for autoimmune diseases showed higher perturbations on disease genes.
• InTiCAR provides new promising insights to *in silico*-based biological network analysis.

## Graphical abstract

**Figure:**
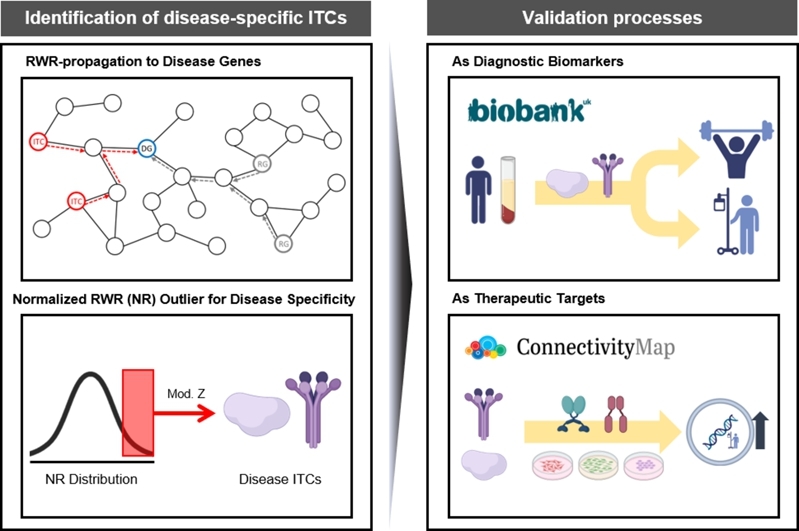

## 1 Introduction

Ever since the first discovery of communication between duodenum and jejunum in 1902 [1], our understanding of the nature and the significance of inter-tissue communication have developed profoundly [2]. These communicators, such as hormones and their receptors, are essential to keep the homeostasis between different parts of the body through a highly interconnected network [3]. Through many intricate interactions within the network, our body can regulate daily bodily processes [4] and respond to external stresses along with the immune system [5].

The causes and the consequences of various disease conditions naturally arise from the disruption of such communication [2]. Increased levels of certain cytokines from specific organs, for instance, have been investigated to drive certain pathological statuses [6], [7], [8]. As such, therapeutic targets aimed at such inter-tissue communicators (ITCs) have been investigated widely by pharmaceutical companies. A recent successful example is the class of GLP-1 agonists, which mimic the GLP-1 from the intestines [9], [10].

Autoimmune diseases (AIDs) represent a diverse group of disorders where the miscommunication results in the immune system's aberrant response to mistakenly attack the body's own tissues and organs [11], [12]. The manifestations of these diseases are clinically classified as either systemic (e.g. lupus erythematosus) or organ-specific (e.g. type 1 diabetes), depending on which part(s) of the body the symptoms take place [11]. There has been an alarmingly steady increase in the global prevalence of AIDs regardless of the sex, age, or ethnicity [13], with approximately one in ten individuals estimated to be affected [13], [14]. Due to the vague symptomatic characteristics of AIDs [15], the diagnosis and the treatment for AIDs still pose significant challenges to overcome [16], [17], [18], [19]. Thus, consistent global efforts on research on AIDs are still required.

Many previous works have suggested *in silico* bioinformatics methods to study AIDs. These studies mostly focused on utilizing expression-based analyses to find differentially expressed genes (DEGs) or network hub proteins, and revealed genes with significant roles in the disease pathology [20], [21], [22]. However, their approaches inherently lacked the means to take inter-tissue communications into account. Considering that AIDs are known to be influenced by various forms of external and internal stimuli [11], [23], [24], an investigative method needs to be developed to seek for ITCs significant for AIDs.

Studying inter-tissue communications through experimental methods has been a daunting task, since *in vitro* tasks only allow simple casual relations to be deduced, while the results from *in vivo* protocols pose difficulty in precise interpretation [25]. A state-of-the-art solution currently under the development is the multi-organ micro-physiological systems (MOMPS) [26], [27], where multiple organ-on-a-chips are interconnected for a simplified anatomic representation of a living body. Despite its promising outlook, the system still requires thorough investigations to properly represent physiology (e.g. cell-to-media volume) [27].

Hence, an *in silico* bioinformatics approach to inter-tissue communications could provide an alternative solution. Accordingly, a number of recent studies has illustrated interesting finding mainly based on three different methods. One delved into the ‘brute force’-based correlations of expression values between all the genes, between all relevant the organs, to find the signaling proteins that have influences on the target organs [28]. The second method involved applying the values from the first method to create a multi-tissue network, where further network propagation-based analyses took place to find the genes significant for signaling [29]. The third involved a construction of disease-specific network with the limited set of known disease genes and their relationships, which may include some ITCs [30], [31]. Despite their meaningful findings, however, the methods used are not suitable in the identification of the key ITCs for diseases of interest. The first two approaches require multi-tissue sampled gene expression values with enough population, but these values are difficult to acquire and are often without the disease context information. The last method requires samples from specific population of patients, but depends on previously known disease genes only, and hence fails to acknowledge the potential relations that disease-specific proteins can have on other proteins.

We therefore propose **InTiCAR**, a network exploration-based analysis to find disease-specific inter-tissue communicators (ITCs) for AIDs, without the need for additional expression data. This was achieved by a normalization process that accentuates the potential relations of ITCs to diseases, through comparisons with random genes. To show that the acquired ITCs are disease-specific, we further illustrate their superior diagnostic capabilities and larger perturbation effects on disease genes using independent datasets. Our goal is to illustrate that inter-tissue communications are crucial aspects for *in silico* studies on AIDs, and that InTiCAR can provide a solid ground to search for relevant ITCs.

## 2 Materials and methods

### 2.1 Overview

The identification of signaling protein molecules that are significant to autoimmune diseases (AIDs) was achieved by using InTiCAR, an *in silico* analysis method applied on biological network. These communicators, which are mainly protein ligands and receptors, are referred to as inter-tissue communicators (ITCs) throughout the manuscript to reflect their potentially diverse roles, whether paracrine or endocrine, in the pathology of the diseases.

**Fig. 1:**
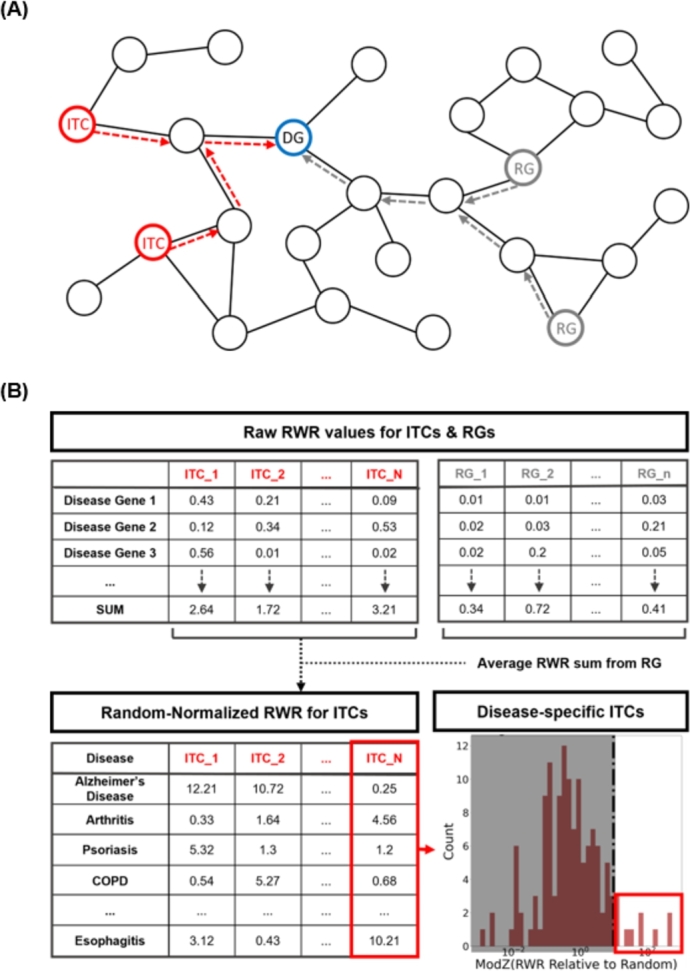
Overview of InTiCAR (A) The schematic diagram of RWR in an *in silico* biological network from disease-specific ITCs to disease genes (B) Simplified illustration of the InTiCAR with example RWR values. Normalization processes of RWR values by random genes (RGs) and by comparison among diseases are shown for disease-specific ITCs acquisition.

**Fig. 2:**
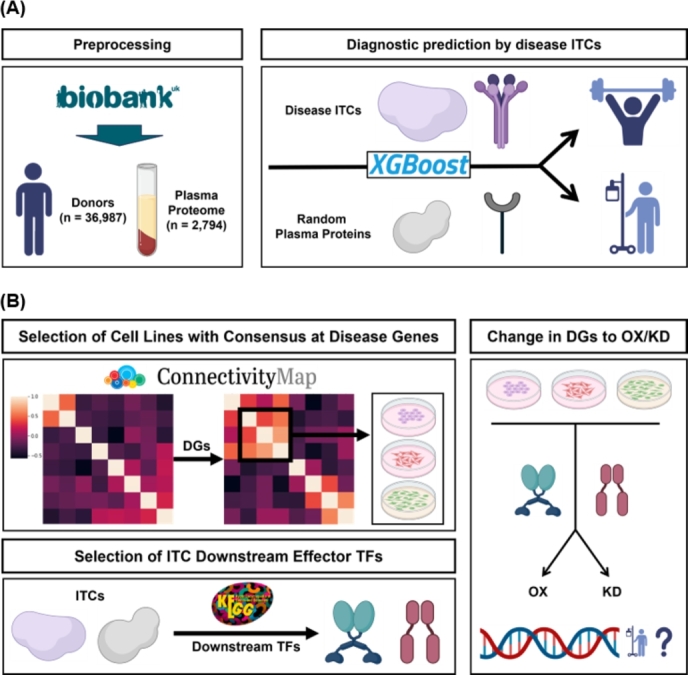
Schematic drawing of the validation process for disease-specific ITCs, (A) as diagnostic markers by using UK Biobank plasma proteome, and (B) as Disease-specific ITCs as therapeutic targets by using CMap perturbation data. Abbr. ITCs=Inter-Tissue Communicators; TFs=Transcription Factors; DGs=Disease Genes; OX=Over-Expression; KD=Knock-Down. (Created with the icons from BioRender.com.)

The following subsections describe the specific steps involved in InTiCAR, as well as the validation processes for resulting disease-specific ITCs. InTiCAR begins with the application of Random-Walk with Restart (RWR) algorithm in a biological network, with ITCs provided as seeds. The normalized RWR values for the disease genes are then acquired to identify disease-specific ITCs (Fig. 1). These ITCs are then validated as either diagnostic biomarkers or therapeutic targets for each disease case (Fig. 2).

### 2.2 Compiling databases for congruous analyses

Several databases were parsed in unified format for integrative analyses involving a biological network, disease-gene relations, as well as inter-tissue communicators.

#### 2.2.1 Construction of biological network

**Figure S1:**
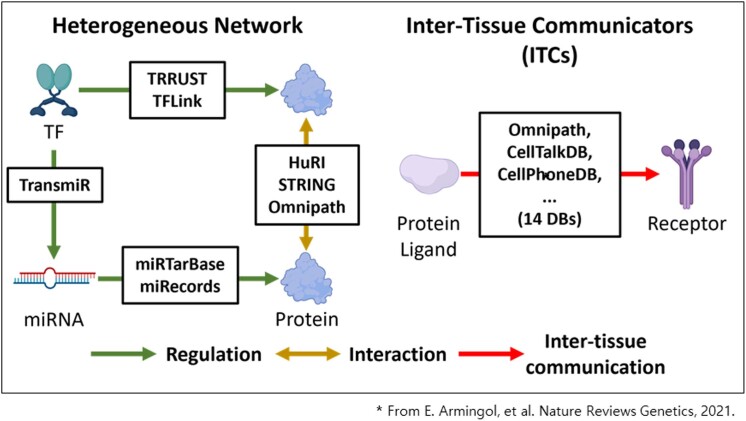
Databases for the biological network used in the study. The edge types and the biological entities are illustrated, along with the names of the source databases. (Created with the icons from BioRender.com.)

InTiCAR requires an *in silico* biological network to run the overall analysis. For this work, we have prepared one using the information based on three major biological interactions: protein-protein interaction (PPI), transcription factor(TF)-target regulation, and microRNA (miRNA)-target regulation (Supplementary Fig. 1). All the edges were acquired from publicly available databases, and only the experimentally validated or manually curated edges were included for the network construction (Supplementary Table 1). The PPI edges were acquired from HuRI [32], STRING [33], and OmniPath [34], all of which combined to 268,700 edges from 15,607 nodes. The TF-target regulation edges, which included regulations on both genes and miRNA, were acquired from TRRUST [35], TFLink [36], TransmiR [37], and OmniPath [34], combined into 206,873 edges from 18,986 nodes. Also, the miRNA-target regulation edges were acquired from miTarBase [38] and miRecords [39], resulting in 9,301 edges from 3,416 nodes. All the biological entities were converted into Ensembl ENSG ID format for successful integration of several types of interaction edges. Lastly, whenever possible, protein complexes were kept as a single node in the network to reflect their behavior *in vivo*. The total of 480,386 edges from 20,598 nodes for the heterogeneous network.

#### 2.2.2 Acquisition of disease genes (DGs)

The DGs for each disease were also acquired from the following public databases: DisGeNET [40], TTD [41], OMIM [42], and DISEASES [43]. As we compiled from several sources with diverse approaches, a disease-gene relation could be disease-related mutations, diagnostic biomarkers, or therapeutic targets. In order to acquire a reliable disease-gene relations, adequately high thresholds were applied based on each database's scoring system (DisGeNET: >0.9 EI; DISEASES: >4 ConfidenceScore). The disease genes were further converted into Ensembl ENSG ID format to match the biological network constructed, and the genes without ENSG ID were discarded. Also, only the diseases whose names can be converted directly into Unified Medical Language System (UMLS) IDs were selected for easier integration with other databases. Lastly, only the diseases that have more than 10 related disease genes were included for the further analysis. This resulted in a total of 265 diseases, including both AIDs and non-AIDs, for the ITC analyses.

#### 2.2.3 Acquisition of inter-tissue communicators (ITCs)

The ITCs were acquired from the set of ligand-receptor (LR) relations parsed from 14 well-known inter-cellular signaling datasets or databases, including OmniPath [34] and CellPhoneDB [44] (Supplementary Fig. 1). The other 12 were directly downloaded from the LewisLab compendium for LR pairs [45]. We carefully selected edges with at least one experimental support, and kept the complexes as a single entity. The final set had 3,437 ligand or receptors. All the ligands and receptors in the final set were used as the ITCs.

### 2.3 Network-based acquisition of disease-specific ITCs

InTiCAR determines the disease-specific ITCs by applying RWR algorithm on a given biological network to calculate the influence of ITCs onto the disease genes, compared to that of random genes in the network. This section clarifies the details involved in each step.

#### 2.3.1 Random walk with restart (RWR)

The RWR is an algorithm, defined and utilized in numerous previous works [46], [47], [48], [49], [50], to measure the extent of relevance between nodes in a given network [51]. This is achieved by following a probabilistic scenario where a particle travel through the network, starting from a given node. The particle can continue its travel to neighboring nodes, but it can also choose to restart the travel from the first node, just like a person surfing in the internet [51]. Depending on the topology of the network, we can calculate the probability distribution of where the particle can be at a given discrete time point t. The formal definition of the RWR is defined as the following:

$$p^{t+1}=(1-r)Wp^{t}+rp^{0}$$

where W is the column-normalized adjacency matrix of the network, while $p^{t}$ refers to the vector whose *i*-th element is the probability of the said traveler being at node *i* at time *t* [49] (see Supplementary File 1 for a detailed description). Such distribution of values can in turn be translated to the level of closeness between the starting node to other nodes. In this research, these RWR values were used to find ITCs that are close to the previously known disease genes.

#### 2.3.2 Acquisition of the threshold RWR value with biological significance

The minimum RWR value required for biological significance in the network were calculated by comparing RWR values between genes in well-known biological processes to those between random genes (RGs).

**Fig. 3:**
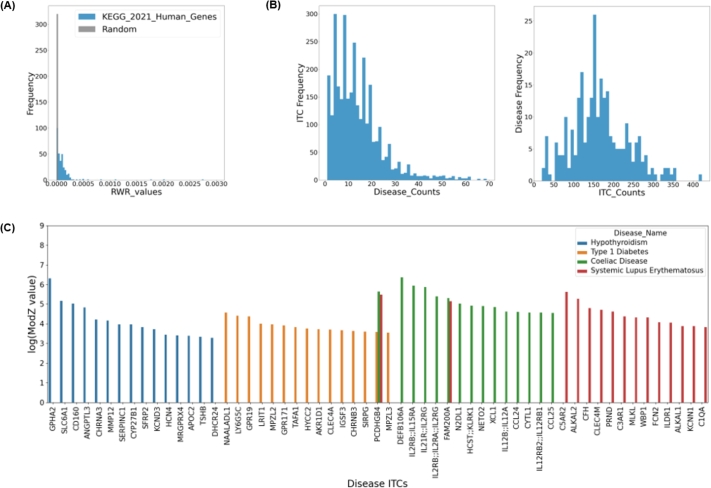
Statistics and examples of disease-specific ITCs. (A) Comparison of RWR values between biologically-relevant KEGG pathway genes and those between random genes (B) (left) Histogram of ITC appearances and (right) Histogram of Disease appearances (C) Top 15 disease-specific ITCs acquired for different AIDs, and their modZ values; only ITCs that were not previously known disease genes are shown.

To achieve this, first, the genes involved in the KEGG pathways [52] were acquired from the EnrichR libraries [53]. For a gene set in each KEGG pathway, RWR was calculated with each gene in the set as a seed. The same number of genes were randomly selected from the network, and RWR was applied between them as well. Then, the median RWR values were acquired from all the KEGG pathways as well as from the RGs for comparison. As the two groups clearly showed differences in values (Fig. 3A), the maximum of the median values from RGs ($2.2884\times 10^{-5}$) was selected as the minimum RWR value required to expect any biological significance.

#### 2.3.3 Computation for disease-specific ITCs

For a given disease, the disease-specific ITCs were computed using the following steps (Fig. 1). Firstly, the RWR was run in the given network with each ITC as a seed node, one ITC at a time. Then, the RWR values on each of the disease genes (DGs) were recorded, if they were over the aforementioned minimum threshold for the biological significance. The overall influence of a given ITC to DGs was finally calculated by summation. The same process, from running RWR to summations at DGs, was repeated for all ITCs, as well as the same number of random genes (RGs). The average RWR values from RGs were used to normalize each ITC's RWR value by division. This process made sure that the ITCs' influence on the DGs also took into account for the edge degrees for the DGs; i.e. hub-like DGs would have high RWR values from any genes.

Then, for each ITC, the RWR values were compared between all available diseases, to make sure that the ITC is specifically related to an outlier subset of diseases only. The modified Z-scores (modZ) were calculated for all diseases for a given ITC, and those above a threshold of 5 were considered specific to the given ITC.

### 2.4 ITCs validation by diagnostic prediction

The diagnostic capability for disease-specific ITCs were tested by using the plasma proteomics dataset from UK Biobank (UKB)-Pharma Proteomics Project (PPP) [54] (Fig. 2A). The UKB-PPP is a collaborative project that collected the 2,923 plasma proteomics dataset from 54,219 donors. As UKB-PPP is a part of the UK Biobank, we were also able to obtain detailed data on each donor, including, but not limited to, physical measurement, lifestyle, imaging, as well as disease status.

#### 2.4.1 Parsing UKB-PPP consortium dataset

The disease status for the donors were acquired from the ‘Diagnoses - ICD10’ field from the UKB datasets. The ICD10 codes were converted into UMLS ID format to match the disease genes or the disease ITCs acquired from the previous steps. Only the diseases whose specific ITCs could be calculated were considered for the diagnostic prediction. Furthermore, only the diseases observed from more than 100 donors were included for the further analyses (Supplementary Table 2).

**Figure S2:**
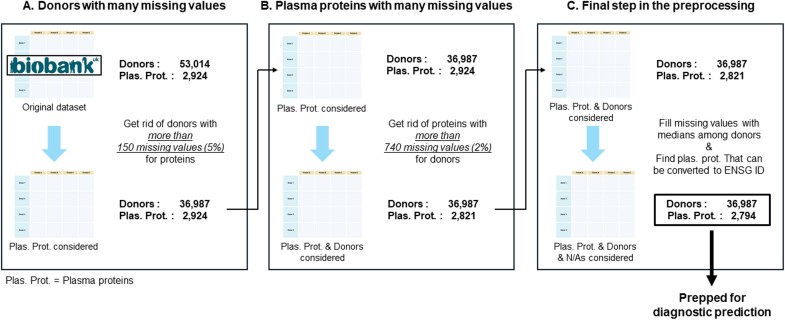
Preprocessing for UKB-PPP dataset for diagnostic prediction. (A) Dealing with donors with many missing values (B) Dealing with plasma proteins with many missing values (C) Final preprocessing to fill in the rest of the missing values. (Created with the icons from BioRender.com.)

The plasma proteome dataset was preprocessed, based on missing values (Supplementary Fig. 2). Every donor had at least one missing data point for plasma proteins, and hence we had to set adequate thresholds, so as to get rid of as many extreme cases as possible, while keeping enough donor cases for modeling diagnostic prediction. The donors with missing values for more than 150 plasma proteins (∼5%) were discarded, leaving with 36,987 donors. Then, the plasma proteins whose values are missing from more than 740 donors (∼2%) were discarded further, leaving with 2,821 plasma proteins. The missing values for plasma proteins were then filled with median values from the rest of the participants. Further conversion of the plasma protein symbols into ENSG ID format resulted in 2,794 plasma proteins from 36,987 donors.

#### 2.4.2 Disease diagnosis prediction model

Each disease case's diagnostic prediction was done by using the XGBoost library [55]. The features used were the disease-specific ITCs, and naturally, only the ITCs that were included for the measurements in the UKB-PPP could be utilized for the prediction. The donors were divided into patients or non-patients, depending on the disease under consideration. As the class distribution was uneven, the ‘scale_pos_weight’ parameter was set as the ratio between the patient and non-patient, so that the model could adjust the weights accordingly.

For the model training, the donors were divided into a training set and a test set, with the ratio of 7:3, by stratified random sampling. The models were trained by 10-fold cross-validation (CV) that are repeated 5 times, using the training set. For each disease, the area under the precision-recall curve (AUPRC) and the area under the receiver operating characteristic (AUROC) were recorded for each model from the CV, by using the hold-out test set.

In order to make sure that the prediction performances achieved by the computed disease-specific ITCs are meaningful, two more features case sets were considered for comparison: random plasma proteins and disease genes. The random features allowed to see whether any plasma proteins in the same number can provide similar performance, while the latter were added to determine whether prior knowledge of disease genes was already informative enough for prediction. In case all the disease genes were not available in the dataset, possibly since they are not ITCs, the prediction score was set to the baseline.

### 2.5 ITCs validation by inferred perturbation

The potential perturbation on disease genes by disease-specific ITCs were acquired by using L1000 Connectivity Map (CMap) Library of Integrated Network-based Cellular Signatures (LINCS) dataset [56] by Broad Institute (Fig. 2B). The L1000 CMap dataset contains a library of gene expression profiles that are obtained from perturbation by ∼5,000 small-molecule compounds and ∼3,000 genetic reagents, across several cell types. L1000 refers to the high-throughput gene expression assay that measures the expression levels of 978 “landmark” genes, whose expression profiles are then used to infer those for 11,350 additional genes. These expression profiles have been useful in identifying the consequential changes that result from over-expression or known-down of specific genes.

#### 2.5.1 Parsing L1000 connectivity map (CMap) perturbation dataset

**Figure S3:**
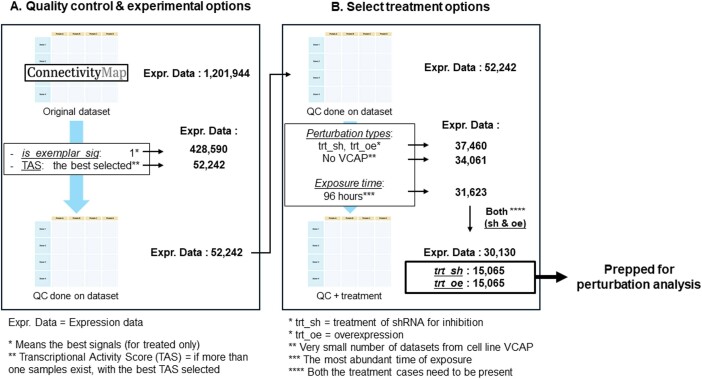
Preprocessing for CMap dataset for perturbation effects. (A) Dealing with quality control values (B) Dealing with the treatment options. (Created with the icons from BioRender.com.)

The level 5 signatures of the ‘expanded CMap LINCS Resource 2020’ were downloaded from the CLUE data library [57]. The datasets with appropriate quality as well as experimental conditions were selected for further analyses (Supplementary Fig. 3). To begin with, for the quality control of the datasets used, only the datasets that are marked as ‘is_exemplar_sig’ were selected. Also, in cases where more than one datasets exist for a perturbation on a cell line, the dataset with the highest transcriptional activity score (TAS) value was selected. Then, for the experimental conditions, only the datasets for over-expression (OX; labeled as ‘trt_oe’) and the knock-down by shRNA (KD; ‘trt_sh’) were collected. Among the collection, the cases whose perturbation time was ‘96 hours’ were further selected, as it was the most abundant condition. The datasets from the cell line ‘VCAP’ were then excluded, as only few were available. This resulted in 30,130 perturbation signatures that cover 8 cell lines, with at least 1,665 perturbations available for each cell line.

#### 2.5.2 Inferring perturbation from correlated cell lines

**Figure S4:**
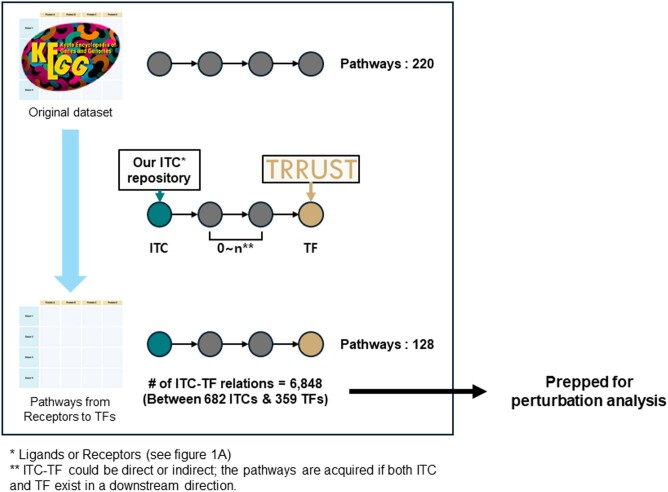
Preprocessing for ITC-TF relations from KEGG. The overall method to link ITCs to TFs based on known KEGG pathways are shown step by step. (Created with the icons from BioRender.com.)

**Figure S5:**
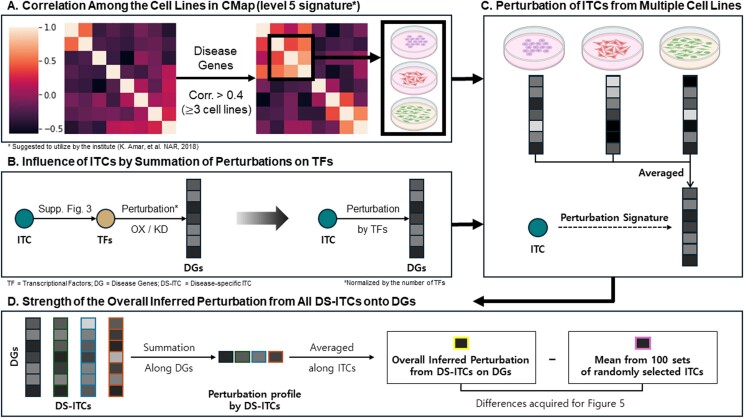
Inferred Perturbation by ITC from CMap. (A) Finding >3 cell lines with high correlations (B) Inferring perturbations by ITCs from those by TFs, normalized by the number of TFs (C) Aggregating perturbation values from multiple cell lines (D) Aggregating perturbation values for multiple disease-specific ITCs into perturbation profile, to compare with random ITCs. (Created with the icons from BioRender.com.)

Since the perturbation data in response to disease-specific ITCs were not directly available from the CMap dataset, we needed to infer the influence of ITCs using their downstream TFs, which were acquired as described previously [58]. Briefly, a list of receptor-TF relations were acquired by searching through the KEGG pathways, and checking if there is any downstream path that leads from a receptor to a TF (Supplementary Fig. 4). A list of ligand-TF relations were additionally acquired by inferring from the ligand-receptor relations. The perturbation results for a disease-specific ITC (either a receptor or a ligand) were therefore defined as the summation of normalized perturbation signatures acquired from the ITC's target TFs (Supplementary Fig. 5B). Hence, the over-expression (OX) of the downstream TFs would be the abundant presence of the corresponding ITC, while the knock-down (KD) of the downstream TFs would be the lack there of.

As the perturbation signatures from different cell lines were uncorrelated, a meaningful perturbation signature for a given ITC could not be acquired by using all of the cell lines at once, as they would cancel each other's signatures out. Therefore, for each disease, a set of cell lines were selected based on their correlations specifically at the disease genes only (Fig. 2B). This implied that the signatures on the disease genes would be higher (or lower) compared to the rest of the genes. Nevertheless, the influence of the disease-specific ITCs could then directly be compared to that of the unrelated ITCs. For this process, the correlation values were first acquired between all the cell lines at the disease genes, and clustered based on their similarity. The largest cluster of at least three cell lines whose average correlation between them are over 0.4 were selected for the given disease (Supplementary Fig. 5A). The diseases whose cell line clusters cannot be acquired were discarded from the analyses. Then, the average of the absolute values of the signatures from the selected cell lines was calculated as the ITC's perturbation signature (Supplementary Fig. 5C).

#### 2.5.3 Strength of the overall inferred perturbation from disease-specific ITCs

In order to show that the influence of the disease-specific ITCs on the disease genes is meaningful, the magnitudes of the perturbation by disease-specific ITCs were compared to those by 100 sets of randomly selected ITCs (Supplementary Fig. 5D). For a given disease, each of its specific ITCs' perturbation signatures were calculated, by the aforementioned method. The perturbation signatures along all the disease genes were summed up to acquire a disease-ITCs-based perturbation profile. The average value within the profile was acquired finally get a value that sums up the overall inferred perturbation of disease-specific ITCs on the disease genes. The overall perturbations by 100 sets of randomly selected ITCs (i.e. non-disease-specific) were also calculated, to show that their influences on the disease genes is not as significant as those by the disease-specific ITCs. The ratios of the DiseaseITCs/RandomITCs were calculated against all the 100 random sets, to find the average fold increase. The significance test was done by the one-sample t-test, where the value 1 for random ITCs is compared to the collection of fold increase values.

## 3 Results

### 3.1 InTiCAR identifies disease-specific ITCs

Given an *in silico* biological network and known disease genes, InTiCAR identifies protein ligands or receptors, collectively referred to as inter-tissue communicators (ITCs), that are specifically significant to each disease (Fig. 3).

#### 3.1.1 RWR-based network exploration to find disease-specific ITCs

Since the biological actions of ITCs involves sending a cascade of signals through a biological network [59], [60], [61], [62], InTiCAR estimates the potential influences of ITCs by using the random-walk with restart (RWR) algorithm, with ITCs as the seed nodes. The RWR values at each node were assumed to represent the influence of the ITCs onto the nodes. For a given disease, the disease-specific ITCs could then be determined by statically determining which ITCs exerted the most influence over the disease-related genes.

Since the raw RWR values themselves represent only the topological characteristics of the network, a threshold of $2.2884\times 10^{-5}$ for RWR values with biological significance was acquired (Fig. 3A; see Materials and Methods). The RWR values lower than the threshold were discarded and replaced with zero to indicate no influence on the node from the ITCs. After normalization by random genes, disease-specific ITCs were acquired based on the modified z-scores (modZ), by comparing the distribution of values between diseases. After all the calculations, on median, an ITC was specifically related to 12 diseases, while each disease had 156 ITCs (Fig. 3B).

Each disease, whether an AID or not, was assigned a diverse set of ITCs that included previously known disease genes, as well as new ITCs that can be suggested for further studies (Supplementary File 2). Some ITCs were unavoidably shared between related or similar disease cases, but the overall landscape of selected ITCs and their modZ values were quite unique to each disease (Fig. 3C).

#### 3.1.2 Literature evidences on disease-specific ITCs for autoimmune disease cases

To see whether the overall disease-specific ITC profiles reflect biologically meaningful and yet distinct relations, we searched for literature-based evidences between the some of the diseases and their predicted ITCs.

Hypothyroidism, for instance, had GPHA2 (glycoprotein hormone subunit alpha2) and TSHB (thyroid stimulating hormone subunit beta) high on the list. Either was not present in the disease genes list, but both are the ligands for TSHR (thyroid stimulating hormone receptor) [63], [64], whose response is directly related to the pathology of the disease. Also, CD160 can be found, and its polymorphisms have been suggested to be related to Grave's disease [65], whose pathology is precisely the opposite of hypothyroidism. Furthermore, the list continues down with ANGPTL3, one of lipoprotein lipase inhibitors. ANGPTL3 was found to be down-regulated by thyroid hormones in a rat model [66], and increased in hypothyroidism patients [67], the evidences which highlight the increased rate of cardiovascular diseases with thyroid dysfunctions [68], [69].

Type 1 diabetes (T1D) contains less obvious, but related ITCs on the top of the list. NAALADL1, a brush border enzyme in intestinal lining, was found to be associated with gut microbiome in T1D subjects, potentially leading to intestinal inflammation [70], [71]. LY6G5C, next on the list, is a lymphocyte antigen-6 family member, whose other loci have been investigated for their roles in cancer cells' survival from the immune system [72]. Also, the gene LY6G5C is located within human leucocyte antigen (HLA) class III region, known to be associated with type 1 and type 2 diabetes [73], [74]. GPR19 is a G-protein coupled receptor whose ligand is andropin, a driver of metabolism and energy expenditure [75], whose level was lowered in diabetics [76], [77]. As such, GPR19 has been studied in the context for metabolic syndromes as well, where its absence leads to glucose intolerance [78], [79] and cardiac dysfunction [80].

One noticeable intersection among the lists is the PCDHGB4 (Protocadherin Gamma Subfamily B4), shared between type 1 diabetes (T1D), systemic lupus erythematosus (SLE) and coeliac disease (CE). The protocadherin is the largest family of cadherins, which are responsible for cell-adhesion. It has been suggested previously that the auto-antibodies for other types of cadherins were found in AID patients, and these antibodies may be the cause for loosening the connections between endothelial cells to cause vascular damages in the case of LE or rheumatoid arthritis (RA) [81]. Similarly, cadherins have long been studied for their involvement in T1D [82], [83] and CE [84], [85]. As protocadherins are expressed highly in the nervous system and known to involve in neurological disorders [86], [87], it is possible that autoimmune-related damages to the nervous system could be due to the change in protocadherins.

Hence, without the need for additional data of context-based expression levels, we were able to acquire lists of disease-specific ITCs for AIDs, by determining their influences onto the disease genes in an *in silico* biological network.

### 3.2 Disease-specific ITCs as diagnostic biomarkers

Once the disease-specific ITCs have been acquired, we first wanted to test whether the presence of these disease-specific ITCs can be used to predict the diagnoses of the corresponding diseases.

#### 3.2.1 Diagnosis prediction using disease-specific ITCs on UK Biobank plasma proteome

Considering that the ITCs are either (i) ligand proteins secreted to the plasma or (ii) receptors who respond to the external ligand proteins, it was necessary to utilize plasma proteome dataset, along with the donors' conditions, such as provided in UK Biobank [54]. After matching the ICD-10 codes from the donors' data to the UMLS ID codes, 52 diseases could be identified and predicted, out of the 265 diseases that we have acquired the specific ITCs for. The protein names in the UK Biobank were converted into ENSG format, so that only the overlap between those and the disease-specific ITCs can be used to predict the disease status of the donors. ITCs that were available for each disease case are organized in the Supplementary File 2. The median number of 33 ITCs were available for the prediction.

There was quite a clear distinction between the diseases that can be predicted by the composition of plasma proteins and those cannot be. Regardless of such cases, the median values for AUC were at 0.528, 0.572, 0.590, for the predictions from raw disease genes (rDGs), random plasma proteins (rPPs), and disease-specific ITCs, respectively. The overall median values were brought down by many of the conditions that may not be predicted through plasma composition, such as migraines. Still, the disease-specific ITCs were significantly better than either of the control cases (p-value: $4.459\times 10^{-4}$ vs rDGs, $1.247\times 10^{-2}$ vs rPPs) (Supplementary Table 3).

#### 3.2.2 Significant improvement in diagnostic prediction results observed with AIDs

For the autoimmune diseases (AIDs), the disease-specific ITCs acquired were able to predict at significantly higher rate, compared to the prior knowledge of disease genes or the random plasma proteins. These diseases were identified by using classification labels in Medical Subject Headings (MeSH) or with manual curation, and included the autoimmune forms of the following diseases: systemic lupus erythematosus (SLE), type 1 diabetes (T1D), alopecia areata (AA), coeliac disease (CD), rheumatoid arthritis (RA), and ulcerative collitis (UC). Other various autoimmune diseases were not available for consideration, due to either low number of disease genes (< 10) or insufficient number of the UK Biobank donors (< 100; see Materials and Methods).

**Fig. 4:**
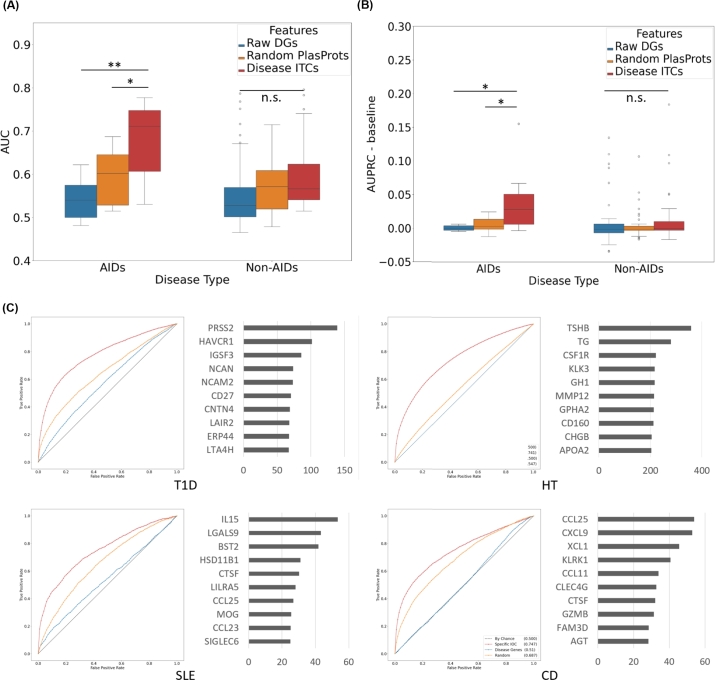
Diagnostic prediction performances using disease-specific ITCs. (A) Comparison of AUCs between different features used for the prediction, as well as between AIDs and non-AIDs. (B) Comparison with AUPRC values per disease (C) AUC results from example AIDs: type 1 diabetes (T1D), hyperthyroidism (HT), coeliac disease (CD), and systemic lupus erythematosus (SLE). ROCs and the top 10 features in the feature importance are illustrated. The color codes for ROC are the same as the boxplots in A above. The values for AUPRC were acquired by subtracting the baseline values for each disease. PlasProts = Plasma proteins; DGs = Disease Genes. IN = IgA nephropathy, HT = Hyperthyroidism, SLE = Systemic lupus erythematosus, CD = Coeliac disease.

**Figure S6:**
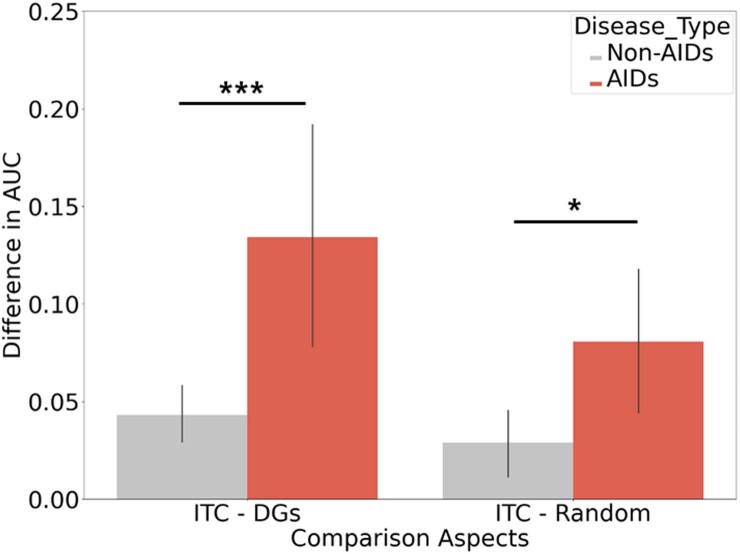
Improvement in AUC by ITCs. Significantly higher improvement by using disease-specific ITCs can be observed with AIDs.

The median AUC values for the AIDs were 0.540, 0.602, and 0.711, for rDGs, rPPs, and disease-specific ITCs, respectively. The differences between the cases were all significant (p-value: $1.272\times 10^{-3}$ vs rDGs, $4.610\times 10^{-2}$ vs rPPs) (Fig. 4A). Such significant difference in the performance could not be observed for the non-AIDs. Furthermore, the extent of improvement performance by using the disease specific-ITCs were significantly higher for AIDs (Supplementary Fig. 6). The comparison of the improved AUC against rDGs and that against rPPs were both significantly higher for disease-specific ITCs (p-value: $1.185\times 10^{-4}$ vs rDGs, $2.084\times 10^{-2}$ vs rPPs), further highlighting the useful additional features acquired.

**Figure S7:**
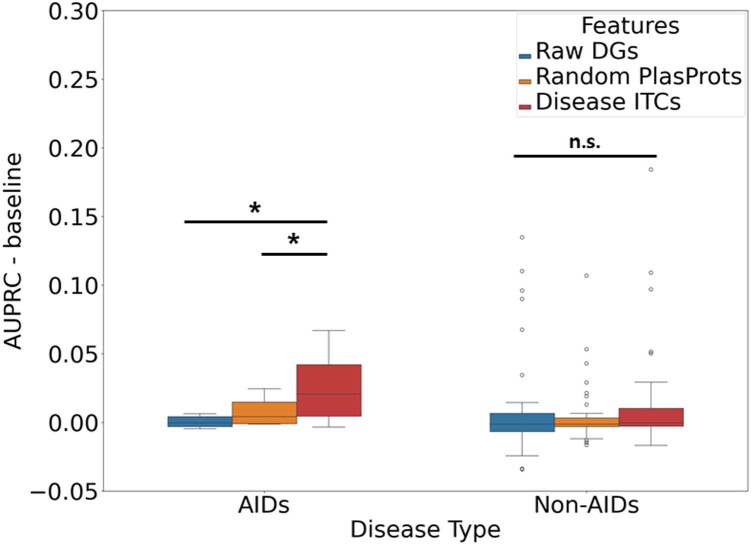
Improvement in AUPRC by ITCs Without Hypothyroidism. The significance test done without hypothyroidism to make sure that a single disease did not drive the significance.

Considering the imbalance between the patients and the non-patient donors, the median AUPRC values were also acquired for the diseases (Fig. 4B, Table 1). Since each disease has a different baseline for AUPRC depending on the ratio of positive datasets available, the differences between the AUPRCs and the corresponding baseline values were acquired for visualization. There was a clear increase in the AUPRC by the disease-specific ITCs, significantly compared to the other two groups (p-value: $1.560\times 10^{-2}$ vs rDGs, $3.081\times 10^{-2}$ vs rPPs). As the most prominent improvement over the baseline was hypothyroidism, it was possible that the significance was solely due to the hypothyroidism. However, the difference was still statistically significant without the disease (Supplementary Fig. 7). It was further noteworthy that, for non-AIDs, the disease-specific ITCs rarely performed the best, or provided meaningful improvement from prior knowledge (rDGs) or chance (rPPs and baseline) (Table 1).

**Table 1:** The top AUPRC values for autoimmune diseases, along with the baselines. The asterisk indicates significance over the random proteins. The numbers in bold indicate the highest performance value among the options.

Autoimmune diseases				
Disease Name	Raw DGs	Random	ITCs	Baseline

Hypothyroidism	0.094	0.082	0.249***	0.094
Type 1 Diabetes	0.015	0.041	0.084***	0.017
Lupus Erythematosus	0.019	0.033	0.065***	0.015
Coeliac Disease	0.007	0.024	0.049***	0.010

Non-autoimmune diseases				

Type 2 Diabetes	0.264	0.236	0.313***	0.129
Chronic ischemic heart disease	0.123	**0.187**	0.182	0.158
Chronic renal disease	**0.157**	0.089	0.155	0.046
Malignant neoplasm of prostate	0.104	0.123	0.138***	**0.138**

Upon the inspection of the features used in the prediction, we were able to determine which of the disease-specific ITCs were utilized in improving the prediction task (Fig. 4C). For type 1 diabetes (T1D), the serine protease 2 (PRSS2) was found to be the top predictor among the T1D-specific ITCs. The gene is known to be highly involved in the chronic or autoimmune pancreatitis [88], [89], [90], which have been recently reported as potential comorbidity for T1D [91], [92]. Interestingly, the serum level of PRSS2 is lower for the T1D patients, as supported by a study from another group [93]. Perhaps the autoimmune conditions in T1D hinder a regulation for PRSS2, causing higher concentration to stay within the pancreas, to eventually cause the pancreatitis. Although pancreatitis was not one of the diseases selected for diagnostic prediction due to the lack of disease genes, a simple hypergeometric test further revealed that the T1D donors in UK Biobank were indeed over-enriched for the pancreatitis (p-value: $6.771\times 10^{-5}$).

For hypothyroidism, the first two features in the disease-specific ITCs were the TSHB (thyroid stimulating hormone beta) and TG (thyroglobulin), both of which were not included in the disease's genes. TSHB is the key factor for the clinical diagnosis for hypothyroidism [94], while TG is the response protein to TSHB that drives the normal physiological behaviors of releasing thyroid hormones [95], [96]. This result further supports the importance of considering inter-tissue communication in the network analysis, and highlights the strength of InTiCAR to uncover useful ITCs important for disease pathophysiology.

For other representative autoimmune diseases as well, the disease-specific ITCs were able to provide features that result in significantly higher performance, especially compared to disease genes. For instance, in systemic lupus erythematosus (SLE), IL15 (interleukin-15) and LGALS9 (galectin-9) were shown as the most useful features in the prediction. The serum level of IL15 for LE was reported to be elevated [97], but its specific involvement to aggravate natural killer cells and cytotoxic CD4+CD28-T Cells in the disease models has only been recently uncovered [98], [99], [100]. LGALS9, on the other hand, is a very recently discovered biomarker for LE [101], [102], [103], [104]. Its roles in the disease, however, are not fully concluded yet, as there are contradicting evidences on the effects of galectin-9 on LE [105].

For Coeliac disease (CE), CCL25 (cytokine ligand 25) and CXCL9 (chemokine ligand 9) were on the top of the features list. The crucial roles of CCL25 and its receptor CCR9 have been validated for the innate immune response's recruitment of the mucosal lymphocytes to the intestine [106], [107], [108]. Their involvements with the immune responses have further been illustrated to the extent of thymocyte development [109], and have also been supported through the observation of CE-related SNPs on these genes' regions [110], [111]. The CXCL9 is also a pleasant surprise to the list, since the chemokine and its receptor CXCR3 are involved in the immune activation [112], and in autoimmune conditions [113], [114]. Especially, the gluten-specific CD4+ T cells are known to express CXCR3, responding to the CXCL9 and CXCL10 that are expressed in the coeliac regions, for which CXCL9 and CXCR are suggested as the therapeutic targets for the coeliac disease [115], [116], [117].

Thus, the InTiCAR-derived disease-specific ITCs can suggest previously known, as well as recently discovered, pathophysiologically meaningful biomarkers, whose presence in the plasma can be used to predict the disease status.

### 3.3 Disease-specific ITCs as therapeutic targets

Once the diagnostic capabilities of the disease-specific ITCs for autoimmune diseases (AIDs) were illustrated, we wanted to test whether we could acquire experimental data-based support for the potential therapeutic influence the disease-specific ITCs.

#### 3.3.1 Inferring the influence of the disease-specific ITCs onto disease genes from CMap LINCS dataset

One of the experimental methods to acquire the perturbation data in response to disease-specific ITCs would be, to observe the change in the gene expression profiles of a disease-representative cell line after the media treatment of the said ITCs. Since we were working with multiple diseases, each with different sets of ITCs, this was not possible to achieve. Hence, we inferred the influence of a set of ITCs on a set of disease genes from CMap LINCS [56].

In short, the perturbations of disease genes by relevant ITCs were estimated from those by downstream transcriptional factors, under the context of multiple cell lines with correlated expression profiles (see Materials and Methods). The justification for this approach was that we needed a solid perturbation signal among the disease genes only, to be at the even ground to compare between the potential influence of the disease-specific ITCs and the non-specific ITCs.

#### 3.3.2 Larger perturbation on disease genes by disease-specific ITCs observed with AIDs

Due to the fact that only the ITCs whose downstream TFs were present in the CMap perturbation could be utilized (Supplementary Fig. 3-5), only the inferred perturbation profiles for 15 AIDs were acquired, which were calculated from, on average, 49 ITCs per disease. The ratios between the perturbation on the disease genes caused by the disease-specific ITCs and those by the 100 random sets of non-specific ITCs were then acquired. Since the result of the perturbation can be positive (up-regulated) or negative (down-regulated) depending on each disease gene, only the absolute values were used to acquire the perturbation to be interpreted as the influence on the disease genes.

**Fig. 5:**
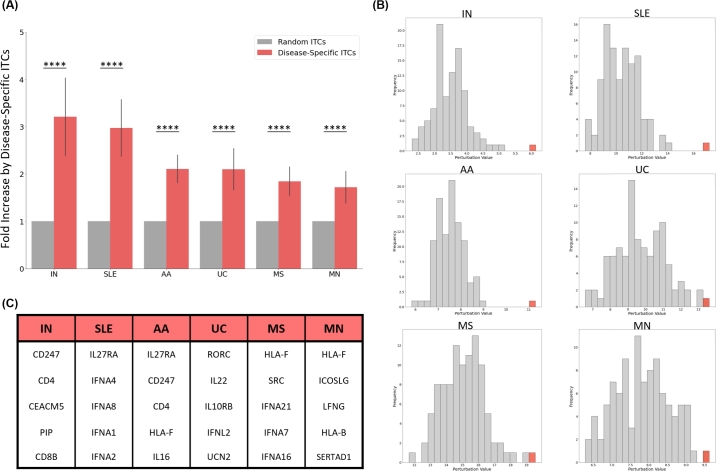
Comparison of the perturbation onto the AID genes by disease-specific ITCs and random ITCs from KD cases. (A) A bar graph showing results from 5 example diseases with the highest perturbation (B) Histogram of median perturbation values for each disease case. Red box indicates the value from the disease-specific ITCs. (C) Examples of disease-specific ITCs with the highest perturbation values for each AID. KD = Knock-down, IN = IgA nephropathy, SLE = Systemic lupus erythematosus, AA = Alopecia areata, UC = Ulcerative colitis, MS = Multiple sclerosis, MN = Membranous nephropathy.

For most AIDs, the extent of perturbation by the disease-specific ITCs was significantly higher compared to random ITCs, as indicated by the fold increase of the expression changes at the disease genes (Fig. 5, Supplementary Fig. 9). This was especially valid for the KD cases, which implied that the KD of the disease-specific ITCs, or that of the downstream TFs, could have an influential impact on the expression profile of the disease genes. Detailed values and statistics can be found in the Supplementary File 3. For KD, the highest perturbation values were observed (all with p-value < $1.0\times 10^{-4}$) for IgA nephropathy (IN; fold increase of 3.21), systemic lupus erythematosus (SLE; 2.97), alopecia areata (AA; 2.11), and ulcerative colitis (UC; 2.10). The fold increase from OX was not as dramatic, but a different subset of AIDs were the most affected (all with p-value < $1.0\times 10^{-4}$): IN (1.79), type 1 diabetes (T1D; 1.69), arthropathic psoriasis (AP; 1.60), and coeliac disease (CD; 1.59). The histograms of the perturbation values further highlighted that the disease-specific ITCs could bring about exceptionally large differences for the disease genes compared to the random ITCs (Fig. 5B).

By examining each of the disease ITC's perturbation values on the disease genes, we were able to ascertain which ITCs were the most significant for the observed change (Fig. 5C). For IN, the highest perturbation level was observed with CD247, which is an integral part of T-cell receptor-CD3 complex [118]. Although the direct evidence that links CD247 to IN could not be found [119], CD247 is known to have a critical role in antigen recognition [118] and intact immune responses [120], [121], [122], and hence is likely to have an important role in autoimmune responses [118]. CD247 was also one of the most influential ITC for AA, and the potential linkage between kidney damage and AA in patients with immune problems have been reported previously [123], [124], [125], which suggests that CD247 could be the key to tackle both cases. The next was followed by CD4, which is a crucial co-receptor for T-cell receptors [126], and involved in binding to antigen presenting cells [127]. It has been reported widely that IN patients have lowered CD4+ cells [128], and that the depletion of CD4+ cells can lead to organ-specific autoimmunity [129], for which T-cells have been suggested as the therapeutic targets for IN [130].

The highest perturbation for SLE was with IL27RA (interleukin 27 receptor subunit alpha), whose ligand IL27 is well-known for its role in stimulating germinal centers (GCs), where high-affinity B-cells mature with the help of T follicular helper cells [131]. Interestingly, there are contradicting evidences as to the roles of IL27 and IL27RA on the development of LE. Some studies find them to be the enhancer of immune and inflammatory response, leading to LE phenotype [132], [133], [134]. Others have found that their presence is required to regulate immune responses and avoid lupus-like symptoms [135], [136]. Moreover, AA also shared IL27RA as one of the top perturbations. The relationship between SLE and AA has been documented and examined through several studies [137], [138], [139], [140], and with our finding, it may be possible that the inflammatory responses from IL27RA could be the source of the comorbidity.

A disease with one of the strongest perturbation fold change observed by OX was T1D (Supplementary Fig. 9). For T1D, the ITC with the highest value was HGF, hepatocyte growth factor (Supplementary File 3). It is noteworthy that HGF has been recently studied as therapeutic targets to attenuate the blood glucose levels [141], protect or regenerate pancreatic beta cells [142], [143], or even improve the engraftment and functions of islet cells [144] in mice. Such findings provide additional support for the disease-specific ITCs found in this study, as well as highlight further the importance of studying inter-tissue communication.

Overall, disease-specific ITCs had higher perturbation on the disease genes for AIDs, and these ITCs could be the sources for potential therapeutic targets, as supported by literature evidences.

## 4 Discussion

Evidenced by numerous high profile studies considering the inter-cellular communications [45], [145], [146], there has been a growing interest in the changes involved in inter-tissue communications under disease conditions [147]. As such, various studies have experimentally validated the influence of specific tissue-based cytokines that bring about symptoms [148], [149]. Hence, deciphering external communications between the cell types and between the specific tissues [148], [149], [150] can provide more precise solutions to treating diseases, in doing so achieve better treatment results and avoid unwanted side effects.

To the best of our knowledge, this is the first *in silico* study that focused on finding such inter-tissue communicators, rather than focusing on the entities in intra-cellular network, which is the usual approach to the network-based disease studies. We instead turned our attention to extra-cellular stimuli that can influence the network, especially at the disease genes. By propagating signals throughout a given biological network, and thereby normalizing the influences of inter-tissue communicators (ITCs), we were able to acquire disease-specific ITCs.

The strength of InTiCAR comes, not only from its unique consideration of inter-tissue communications, but also from that it did not require any expression datasets under autoimmune disease contexts to find the disease-relevant ITCs. The process can be applied to any biological network, for any disease with previously known genes. More impressively, the predicted ITCs were shown to have promising usages in either diagnostic or therapeutic purposes on renowned external datasets, such as UK Biobank and CMap LINCS. The literature-based validation further highlighted the important roles that ITCs hold in regulating or influencing the immune responses at various levels. Hence, InTiCAR was able to capture the crucial inter-tissue communication between the blood and the relevant target organs. The top 5 ITCs that were supported for AIDs from each validation process are organized in the Table 2.

**Table 2:** The top 5 lists of disease-specific ITCs for examples of AIDs from this study. The criteria for inclusion are: if a disease was eligible for each validation task, the diagnosis AUC of at least 0.65 that is significantly higher than AUC for raw disease genes, and significantly higher perturbation on disease genes observed for the disease-specific ITCs compared to random ITCs.

Validated disease-specific ITCs		
Disease Name	Diagnostic Biomarkers	Therapeutic Targets

Type 1 Diabetes	PRSS2, HAVCR1, IGSF3, NCAN, NCAM2	HGF, IL6, GREM2, LIPA, ACVR1C
Lupus Erythematosus	IL15, LGALS9, BST9, HSD11B1, CTSF	IL27RA, IFNA, IFNK, PDCD1LG2, ICOSLG
Hypothyroidism	TSHB, TG, CSF1R, KLK3, GH1	-
Coealic Disease	CCL25, CXCL9, XCL1, KLRK1, CCL11	-
IgA Nephropathy	-	CD247, CD4, CEACM5, PIP, CD8B
Alopecia Areata	-	IL27RA, CD247, CD4, HLA-F, IL16
Ulcerative Colitis	-	RORC, IL22, IL10RB, IFNL2, UCN2
Multiple Sclerosis	-	HLA-F, SRC, IFNA, INFK, IL36G

It is important to note that the AIDs-specific ITCs illustrated significant improvement in the diagnostic prediction, compared to the prior knowledge of disease genes. This was in part because many of the disease genes were not found in the plasma. This finding implies that the current understanding of the immune diseases clearly lacks a crucial aspect of the diseases to consider, which is the inter-tissue communications. Considering that the development of AIDs involves a complex cascade of immune responses to various stimuli [151], [152], and that ITCs like hormones have been suggested to play critical roles in AIDs [23], [153], [154], it is crucial to continuously develop methods that can utilize ITCs in the investigations.

The unexpected high performance by random plasma proteins in the diagnostic prediction is also noteworthy. Various biobanks have recently released proteomics data for researchers to investigate [54], [155], and many studies have already confirmed and suggested the power of plasma proteins as meaningful biomarkers [54], [155], [156], [157]. Considering that blood proteomics have been shown to be rich in biological meanings, it might not be surprising that a set of random plasma proteins may be enough for a diagnosis. Hence, about 34 randomly chosen plasma proteins (i.e. the average of the available disease-specific ITCs) could be sufficient to provide somewhat meaningful predictions, although still significantly lower than the disease-specific ITCs.

The particularly effective performance by ITCs for AIDs could be attributed to the complex cascade of immune responses to external stimuli [151], [152]. The *in silico* biological network is known to reflect both small-world and scale-free nature [158], [159], and perhaps utilizing RWR in such network could have driven the immune-related inter-tissue communicators to have stronger impact on the disease genes. As such, one way to apply the suggested RWR analysis to other diseases could be to construct the disease-specific network whose components reflect the genes that are most likely expressed in the disease-related tissues.

**Figure S8:**
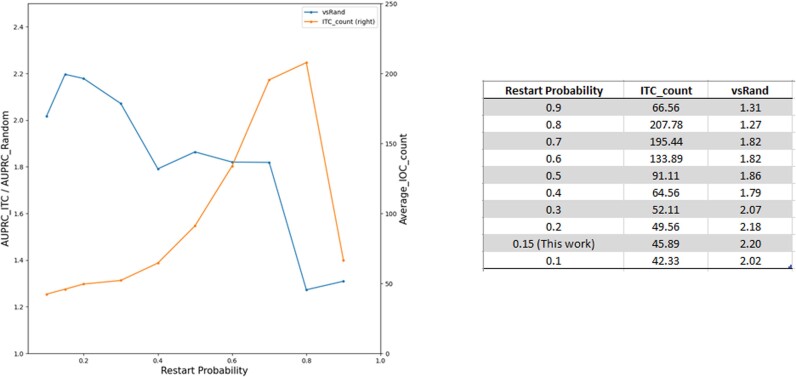
Changing Restart Probability. The diagnostic prediction results for the autoimmune diseases, depending on different disease-specific ITCs acquired from varying the restart probability during RWR. The probability 0.15 has the best increase in the AUPRC compared to random, using a small number of features.

**Figure S9:**
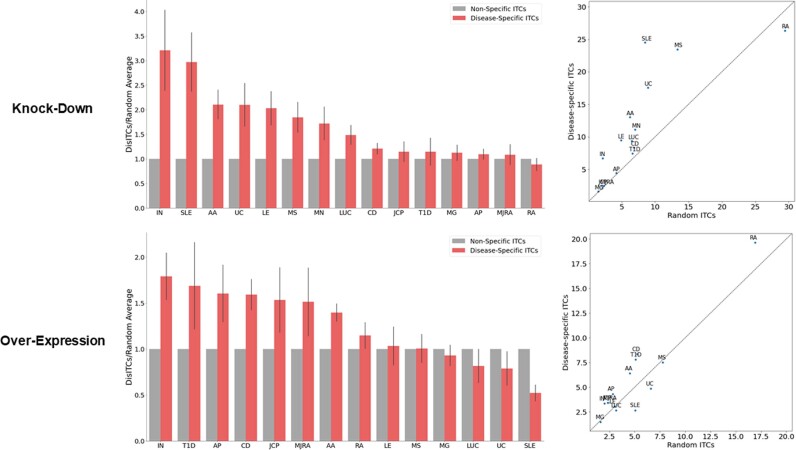
Ratio Disease Specific ITCs over Random Sets. The overall perturbation results acquire for AIDs from CMap, with knock-down and over-expression. (Left) Bar graph showing the fold-increase perturbation change with disease-specific ITCs (Right) Scatter plot showing which diseases had stronger responses with disease-specific ITCs, by comparing to the line y=x.

The random walk with restart (RWR) algorithm used in this study allowed an effective search for ITCs that could have significant impact on the disease genes. This is significant in that the only materials used for the search were the network and the prior knowledge of disease genes. Changing the restart probability for the algorithm had an interesting impact on the number of disease-specific ITCs, as well as the performance of the diagnostic prediction (Supplementary Fig. 8). The increase in the probability increased in the number of disease-specific ITCs, but reduced the diagnostic prediction significantly. This implies that focusing on the edges near the seed nodes prevented the detection of disease-specific ITCs. For the network used in this study, the well-known community standard of 0.15 [160], [161] was shown to be the most effective in finding ITCs.

From this study, the currently available set of prior knowledge disease genes from publicly available databases was utilized to find significant disease-related (Fig. 3). This implies that, even when only limited number of disease genes are available for research, InTiCAR can find useful therapeutic inter-cellular communicators. It is necessary to mention one of such example, which was TSHB for hypothyroidism. We were surprised at its absence in the disease list for hypothyroidism, as it is an obvious major player for the disease. It turned out that TSHB was present in the disease genes dataset from OMIM, but only for a very specific subtype of hypothyroidism, named ‘hypothyroidism, congenital, nongoitrous 4’, which was assigned to UMLS ID of ‘C0271789’, other than the general “hypothyroidism” followed throughout the study, whose ID was ‘C0020676’. As we were dealing with a pan-disease analysis, it was not feasible to manually curate the diseases, or simply assign a set of seemingly related diseases to a single disease with subjective criteria. Hence, our approach in the acquisition of disease genes was limited to strictly following the UMLS ID system, which explains the absence. The noteworthy point is, however, that the very protein could still be recovered with InTiCAR.

Lastly, we have implemented a Python tool for any interested researchers to run InTiCAR on their local machines. The tool takes any *in silico* biological network, along with any set of genes-of-interest, to calculate and determine the ITCs that are the most relevant. The repository for InTiCAR is publicly available at https://github.com/kwnskim/InTiCAR.git. The detailed user guide for the tool can be found in the Supplementary File 4. We hope that this study provides the basis for the *in silico* network-based research into the inter-cellular and inter-tissue communication under disease context.

## Funding

This work was supported by the 10.13039/501100014188Ministry of Science and ICT through the National Research Foundation (RS-2023-00262747). This funding source had no role in study design, data collection and analysis, decision to publish, or preparation of the manuscript.

## CRediT authorship contribution statement

**Kwansoo Kim:** Writing – review & editing, Writing – original draft, Visualization, Validation, Software, Project administration, Methodology, Investigation, Formal analysis, Data curation, Conceptualization. **Manyoung Han:** Writing – review & editing, Visualization, Resources, Investigation. **Doheon Lee:** Writing – review & editing, Writing – original draft, Supervision, Resources, Project administration, Funding acquisition, Conceptualization.

## Declaration of Competing Interest

The authors declare that they have no conflict of interest.

## Data Availability

The datasets used in the current research are available from the corresponding author on reasonable request.

## Appendix A Supplementary material

MMC 1 Statistics on databases for heterogeneous network. The numbers of nodes and edges for all the sources used depending on the interaction types used are organized. MMC 1

MMC 2 UKB-PPP donors per disease. The demographic statistics of the donors for each disease in the UKB-PPP dataset are organized. MMC 2

MMC 3 UKB-PPP Diagnostic Prediction Results. The overall diagnostic prediction results are organized, in terms of AUC and AUPRC, as well as p-value differences between features used. MMC 3

MMC 4 RWR. A document with detailed explanations for the RWR process, as well as the values used in the study. MMC 4

MMC 5 Disease-specific-ITCs. A collection of disease-specific ITCs, with an additional column to indicate whether the ITCs are available in UKB-PPP. MMC 5

MMC 6 CMAP Perturbation by Disease ITCs. A collection of disease-specific ITCs perturbation (knock-down or over-expression) results for AIDs, from CMap. MMC 6

MMC 7 Manual for the Python-implemented tool version of InTiCAR. MMC 7
