# Supplementary material for: InTiCAR: Network-based identification of significant inter-tissue communicators for autoimmune diseases
Source: Comput Struct Biotechnol J. 2025 Jan 10;27:333–45. doi: 10.1016/j.csbj.2025.01.003 (PMC11782887; doi:10.1016/j.csbj.2025.01.003)
Supplement: MMC 4 — RWR. A document with detailed explanations for the RWR process, as well as the values used in the study. [file mmc13.docx]

**Supplementary File 1**

**< Random Walk with Restart >**

The RWR algorithm simulates a random walker traveling from one node to another in a given network. The walker initially travels from a given starting node to available neighbors and follows along the further available edges. The walker is also assumed to start back, with certain probability, from the initial starting node. Each step in RWR represents the set of probabilities that the walker could be at each node. Since such values would represent how closely and efficiently the nodes are connected in the network, many studies have utilized the algorithm to find the influence of the node-of-interest to the rest of the network.

Now, from the formula for RWR:


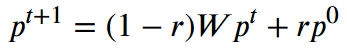


p^0^ is a binary vector where only the starting node (an ITC in our case) is 1, while all other nodes are 0, and it represents the initial probability state of the given network.

p^t^ represents the probability state at the time point t. This would start as p^0^ in the beginning.

r represents the probability that the walker starts back from the initial node. Our restart probability was set to 0.15, which was selected, as it is a value widely used in the random walk models [1,2]. The value would make sure for the walker to travel thoroughly in the network, as well as strengthen the signal from the starting node.

W is the normalized adjacency matrix whose elements represent the presence of edges between the nodes. It is a NxN matrix where N represents the number of all the nodes. For given nodes i and j, the value at indices (i, j; i.e. W_ij_) would be non-zero if there is an edge between them. For an adjacency matrix without normalization, the values would simply be binary to indicate the presence of edges.

Now, the normalization process is done to take into account the number of outgoing nodes for a given node. A random walker at a node i can head towards all k available edges, so all the k neighbors (in the column i of the adjacency matrix) are normalized to have the value 1/k.

The stopping point for the iterative processes in RWR is reached when there is small difference between the states p^t+1^ and pt; the probability values did not change even after a random walk. Specifically, we add all the values in (p^t+1^-p^t^) to acquire the added differences and see if the overall difference is lower than 1.0e-6, which is a threshold used in very similar algorithms like PageRank [1, 3]. There is a very small chance where this convergence cannot be reached, and if the iteration t goes over 100, the algorithm is set to stop and raise an error. This, however, did not happen in our study.

[1] Brin, Sergey, and Lawrence Page. "The anatomy of a large-scale hypertextual web search engine." *Computer networks and ISDN systems* 30.1-7 (1998): 107-117.

[2] Yang, Chao, et al. "Analyzing spammers' social networks for fun and profit: a case study of cyber criminal ecosystem on twitter." Proceedings of the 21st international conference on World Wide Web. 2012.

[3] Köhler, Sebastian, et al. "Walking the interactome for prioritization of candidate disease genes." The American Journal of Human Genetics 82.4 (2008): 949-958.
