# Supplementary material for: InTiCAR: Network-based identification of significant inter-tissue communicators for autoimmune diseases
Source: Comput Struct Biotechnol J. 2025 Jan 10;27:333–45. doi: 10.1016/j.csbj.2025.01.003 (PMC11782887; doi:10.1016/j.csbj.2025.01.003)
Supplement: MMC 7 — Manual for the Python-implemented tool version of InTiCAR. [file mmc16.docx]

**Supplementary File 4**

**< Manual for the tool version of InTiCAR >**

We built a Python tool for other interested researchers to run InTiCAR in local machines. As mentioned in the main text, the repository for the tool is available in the following URL: <https://github.com/kwnskim/InTiCAR>.

The tool was designed so that users with an *in silico* biological network of their choice can find ITCs relevant to any list of genes that the users are interested in. For easier access, the tool can be simply executed at terminal with the user-specified parameters. In addition, the required packages were set so that the minimum criteria (Python==3.8.18; pandas==1.5.3; scipy==1.10.1; numpy==1.24.3; networkx==3.1) would be required to run the code.

The inputs for the tool are the following:

1. **'background_network' (-b)**
   The network to run the analysis from. All the gene pairs need to be in ENSG IDs.
2. **disease_genes_of_interest (-g)**
   A list of genes that a user would like to search the related ITCs for.
3. **disease_genes_full_collection (-d)**
   A dataframe with the prior knowledge-based disease genes. We recommend using the file provided in the repository. This table will provide the reference to compare when calculating the modified Z score for the user’s genes-of-interest.
4. **modified_z_threshold (-t)**
   The threshold to use for the modified z-score to find ITCs specific to the user’s genes-of-interest.
5. **parallel_num (-p)**

A number of cores to use for the parallel processing. Parallel processing is highly recommended.

The example command to run InTiCAR is the following:

**./run_inticar.py -b {dir_for_network} -g {dir_for_gene_of_interest} -p 40**

Any results will be created in the subdirectory named ‘results’, ending eventually to create a CSV file with a table that shows the ITCs with high modified Z score in the descending order.
